# Quantitative Preterm EEG Analysis: The Need for Caution in Using Modern Data Science Techniques

**DOI:** 10.3389/fped.2019.00174

**Published:** 2019-05-03

**Authors:** John M. O'Toole, Geraldine B. Boylan

**Affiliations:** Department of Paediatrics and Child Health, INFANT Research Centre, University College Cork, Cork, Ireland

**Keywords:** EEG, aEEG, preterm, newborn, quantitative analysis, transition, maturation, spectral power

## Abstract

Hemodynamic changes during neonatal transition increase the vulnerability of the preterm brain to injury. Real-time monitoring of brain function during this period would help identify the immediate impact of these changes on the brain. Neonatal EEG provides detailed real-time information about newborn brain function but can be difficult to interpret for non-experts; preterm neonatal EEG poses even greater challenges. An objective quantitative measure of preterm brain health would be invaluable during neonatal transition to help guide supportive care and ultimately protect the brain. Appropriate quantitative measures of preterm EEG must be calculated and care needs to be taken when applying the many techniques available for this task in the era of modern data science. This review provides valuable information about the factors that influence quantitative EEG analysis and describes the common pitfalls. Careful feature selection is required and attention must be paid to behavioral state given the variations encountered in newborn EEG during different states. Finally, the detrimental influence of artifacts on quantitative EEG analysis is illustrated.

## 1. Introduction

Postnatal adaption presents many challenges for the preterm infant, with hemodynamic changes increasing the risk of brain injury. An immature cardiovascular system may not be able to maintain hemodynamic stability, resulting in injuries such as peri/intra-ventricular hemorrhage with associated adverse outcomes ranging from neurodevelopmental delay to death (1). It is therefore important to monitor preterm brain function during the transition period. EEG is the only cot-side tool available in the neonatal intensive care unit (NICU) to do this effectively, as developing brain injury manifests as changes to baseline EEG (2–8)

Multichannel neonatal EEG contains complex spatiotemporal information that can be difficult to interpret, especially for non-EEG experts. In preterm infants, the EEG develops in the most immature neonates at 23/24 weeks' gestational age through to full term age with three major trends: increasing continuity, with defined periods of normal EEG quiescence for specific gestational ages; appearance of several normal transient waveforms of prematurity; and the appearance of sleep cycling. Assessment of an infant's EEG against these parameters can indicate whether the maturity of the brain is appropriate for gestational age. In addition, serial EEGs starting soon after birth have been shown to be of use in both determining the timing and severity of brain injury and for outcome prognosis (9). Therefore, the EEG is critical for diagnosis, treatment, and prognosis in the newborn period.

**Figure 1:**
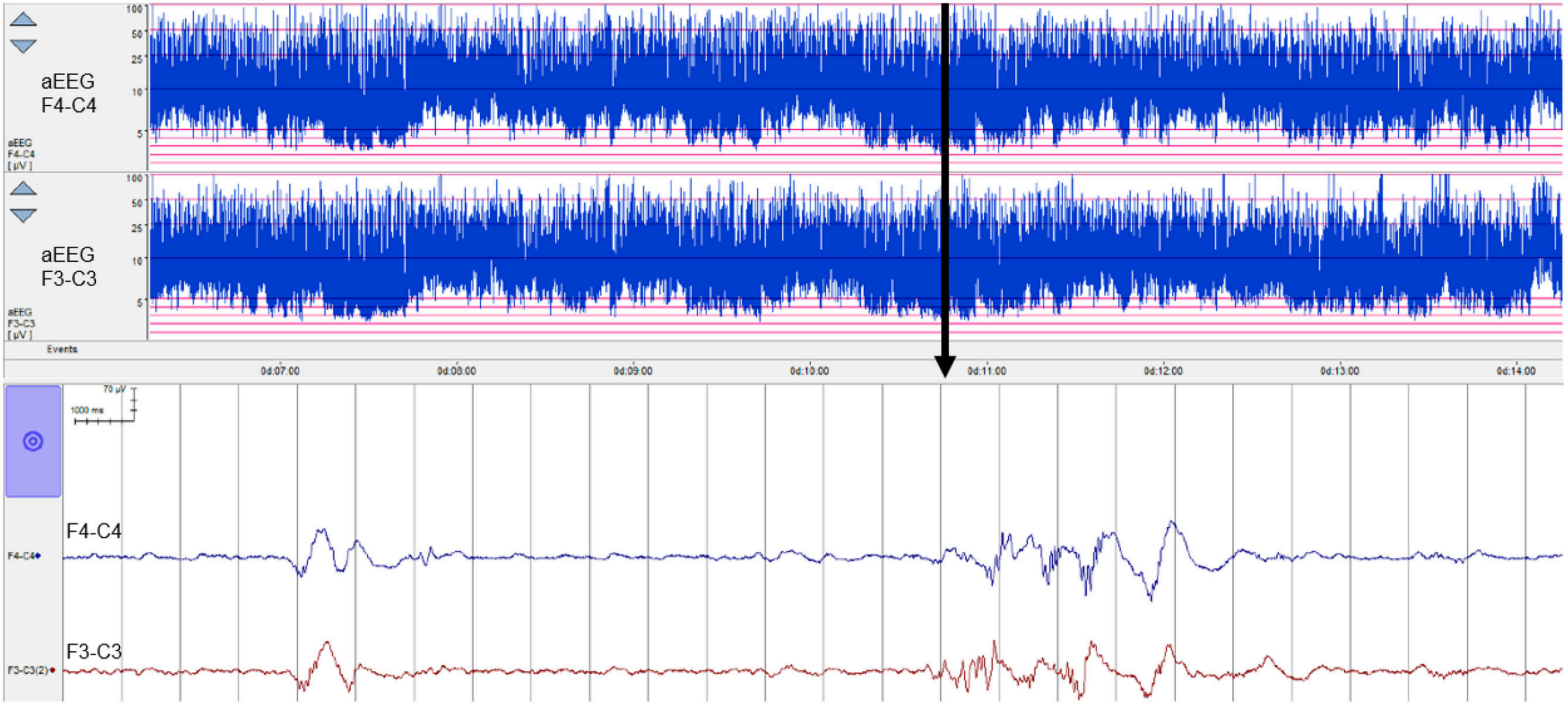
Eight hours of aEEG from a preterm neonate (28 weeks' gestational age) from 6 h of birth. Top two channels show the aEEG from the right and left fronto-central region of the brain and the bottom two channels show 26 s of raw EEG for a point in time marked with an arrow. The aEEG is a compressed EEG signal that has been heavily filtered and then displayed on a semi-log scale. Short duration features of the EEG are impossible to recognize but overall trends are evident.

Experts with the level of experience required to interpret neonatal EEG are scarce and are certainly not always available when this information is needed, which, in the NICU, can be at any time, day or night. As a result, in many NICUs around the world, a simpler EEG method, using a restricted number of spatial channels (1–2 channels) and a compressed EEG display has been adopted—the amplitude integrated EEG or aEEG, see example in Figure 1. aEEG was developed in the 1960s for monitoring comatose adults in intensive care and was never intended for monitoring the neonatal brain. However, it was launched into a technology vacuum and adopted rapidly by NICUs for use in full-term infants in the late 1980s because there was simply nothing else available to provide the much-needed insight into newborn brain function. Over the years, this device has proved very useful in the hands of experienced users, and digital machines now also provide 2 channels of uncompressed raw EEG data as output (Figure 1). Non-experts still struggle to interpret the patterns produced and to distinguish seizures from noisy signals caused by excessive biological or environmental interference (10, 11). The aEEG is essentially just one way to represent the EEG quantitatively and has been used extensively in preterm infants (including during the transitional period) and scores of aEEG maturation have also been devised. However, this has happened gradually without any thorough evaluation of the aEEG as an appropriate tool for the assessment of preterm brain function. In addition, other forms of quantitative analysis of the preterm EEG are now being used, as outlined in the next section.

The aim of this paper is to provide an overview of quantitative measures for the analysis of neonatal EEG that can be used to assess neonatal brain function during transition and to highlight the challenges that can be encountered from both a neurophysiological and signal processing perspective. The measures derived from the EEG using quantitative methods are only of use if there is careful data preparation and management prior to analysis but these steps are easy to overlook in the current era of powerful data science techniques.

## 2. Quantitative EEG Analysis

Quantitative EEG (qEEG) represents an alternative to the visual interpretation of the EEG. This quantitative approach has the potential to replace the complex, time-consuming, and subjective process of the human interpretation of the EEG with a simple, quick, and reproducible computer-based approach. As a computer requires clear instructions, a mathematical approach is needed to compute a characteristic of the EEG. This characteristic, known as a *feature*, is a mathematical summary of the EEG which reduces 1 epoch (time period) of EEG into 1 number. For example, if we calculate the standard deviation of the EEG voltage for the two 40-s EEG epochs in Figure 3, we find that this feature, averaged over the 6 EEG channels, is 37 μ*V* in Figure 3A and 53 μ*V* in Figure 3B. Thus, we have reduced the 40-s epoch, across the 6-channels, to 1 number. The term qEEG typically refers to a collection, or set, of these features. This set can include a diverse range of statistical and signal processing features, from the simple, such as the standard-deviation of the EEG, to the more complex, such as the nonlinear, time-varying connectivity among the EEG channels.

Quantitative EEG has been used extensively in preterm EEG analysis. For example, for preterm infants <32 weeks of gestation, qEEG has been shown to mirror maturation (12–26) and has been used to identify and quantify a temporal evolution of the EEG after birth (27–30). Quantitative EEG has also been used to detect bursts within the EEG (23, 31–39), detect early brain injury (7), and to predict neurodevelopmental outcomes (29, 40–42). However, the real power of qEEG may lie in coupling the feature set with machine learning methods to develop specific classification and regression algorithms. These algorithms are trained and tested on human annotations of the EEG. Recent developments in this area include regression algorithms for estimating the functional maturation of cortical activity (30, 43–45) and classification algorithms for determining sleep cycles (46–48).

**Figure 2:**
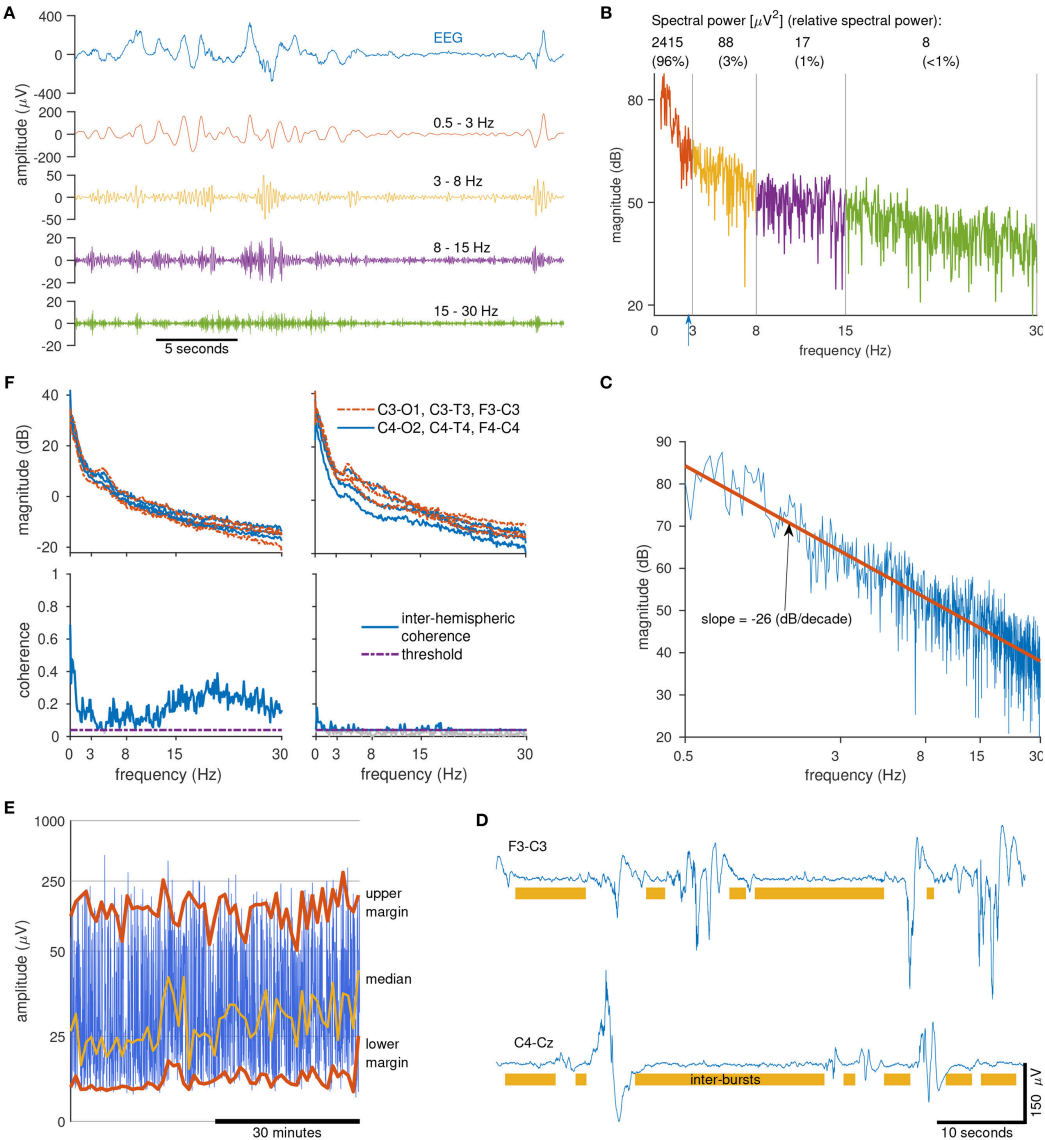
Calculating quantitative EEG features. Clockwise from top left **(A)**: sample of 1 channel of EEG (top) filtered into 4 frequency bands (below). Note the low-amplitude of the higher frequency bands (>15 Hz). **(B)** Spectrum of the EEG signal in **(A)** in decibels. Includes spectral power values and relative-spectral power for each frequency band. Blue arrow at 2.7 Hz marks the spectral edge frequency, indicating that 95% of the signal power is contained within 0–2.7 Hz. **(C)** Log-log plot of the spectrum of the EEG signal from **(A)**. Fractal dimension is a measure of the slope of the line (red line) fitted to the spectrum. **(D)** Two epochs of EEG (from 2 preterms) with annotated inter-bursts (the area not annotated is considered as a burst). Burst/inter-bursts annotation generated from a preterm burst-detector algorithm. **(E)** Range-EEG (rEEG) for 1 h of EEG for 1 channel with median, lower-, and upper-margins highlighted. **(F)** Inter-hemispheric coherence (bottom) estimated from 10 min of EEG (spectra, top) from 2 preterm infants (left and right).

We now present examples of qEEG features used in this report. Probably the most common qEEG feature is spectral power. This feature describes the average power for different frequency bands. Suitable bands for preterm EEG are 0.5–3, 3–8, 8–15, and 15–30 Hz (30, 39, 49). An example of these different frequencies are presented in Figure 2A, where a filter splits the EEG into the four frequency bands. The higher frequencies comprise of the faster oscillations whereas the lower frequencies comprise of the slower oscillations. Figure 2B shows the spectrum of the same EEG signal, plotted in decibels (dB) to highlight the contribution of the higher frequencies. (Without this logarithmic plot it is difficult to visually assess the contribution of the higher frequencies because they are of such low power.) Also included in Figure 2B is the spectral power values (in units of μ*V*^2^) for each frequency band. Relative spectral power, another qEEG feature, is the proportion of power in each frequency band. For our example in Figure 2B, we find that 95.6% of the spectral power is concentrated in the 0.5–3 Hz band, compared to just 0.3% of the spectral power in the 15–30 Hz band. And the last frequency-related feature presented here is the fractal dimension. This feature captures a measure of the shape of the frequency spectrum. If we plot the spectrum on a log–log scale and then fit a line to this plot, the slope of the line is proportional to the fractal dimension (50, 51). Figure 2C shows an example of this, where we find that the slope of the fitted line, −26 dB/decades, equates to a fractal dimension of 1.2 (50, 51).

Other qEEG features are estimated directly on the time-domain EEG signal. An example of this is the maximum duration of the inter-burst interval (IBI). To distinguish the bursts from the inter-bursts, we use an automated method to detect the inter-bursts (39). Everything not detected as inter-burst is classified as bursts, which may include continuous EEG activity (39). Figure 2D shows an example of the annotations generated by this automated method. From this burst/inter-burst annotation, we then generate quantitative features such as maximum IBI or percentage of bursts. Next, to capture a long-duration trend of the EEG we use the range-EEG (rEEG) (18, 38). This was proposed as an alternative to the aEEG because the aEEG—despite its widespread use—is lacking a common definition and will differ from EEG machine to EEG machine. From the rEEG, we estimate trends such as the median, lower- and upper-margins as plotted in example in Figure 2E, and then average over time to generate the feature.

And finally, to quantify some form of connectivity between brain regions, a simple and robust approach is to quantify inter-hemispheric connectivity using EEG coherence. This approach summarizes global connectivity by estimating the coherence between all channel pairs on the 2 hemispheres, for example between F3-C3 and F4-C4, C3-T3, and C4-T4, and so on. Figure 2F shows 2 examples for 2 preterm infants, one with a higher level of coherence (left) comparative to the other (right). After coherence is estimated between channel pairs, a global average (median value) is generated as the frequency-dependent inter-hemispheric coherence. This coherence value is then summarized by taking the average for each of the four frequency bands, similar to the process for spectral power. The threshold in Figure 2F eliminates spurious coherence values by re-defining the zero-coherence level (52).

Before implementing qEEG analysis, it is important to consider a number of factors. First, the selected epoch must be representative of the EEG record, as different EEG activity states influence the qEEG. Second, the feature set is dependant on the application. And third, the EEG must be free from artifacts, otherwise this will distort the analyses. Therefore, it is important to consider the different activity states, before analysis. To highlight the seriousness of these points, we next present 3 qEEG examples generated from different cohorts of newborn EEG. The qEEG features are generated using NEURAL [a neonatal EEG feature set in Matlab, version 0.3.3 (53)]; full implementation details can be found in O'Toole and Boylan (54). We also recommend a procedural approach for qEEG analysis to avoid common pitfalls.

## 3. Example 1: Quantitative Features to Capture Different EEG States

**Figure 3:**
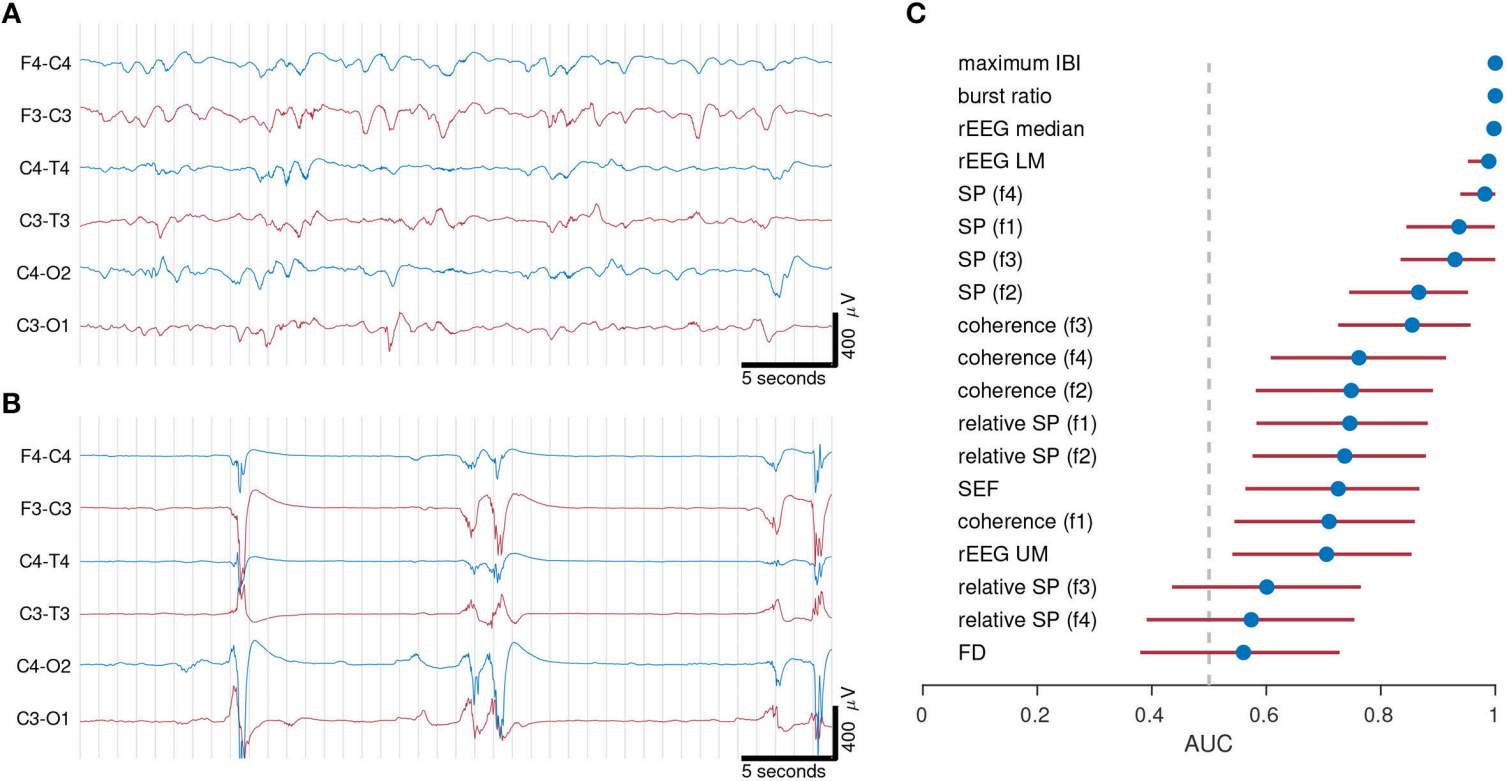
Examples of continuous **(A)** and discontinuous **(B)** EEG activity from 2 preterm infants of gestational age 30 weeks 0 days in **(A)** and 27 weeks 0 days in **(B)**. Quantitative features in **(C)** distinguish between continuous and discontinuous EEG activity. EEG from 21 infants recorded within days after birth. Dots represents AUC (area under the receiver operator characteristic) and lines represent 95% confidence intervals. AUC values of 0.5 represents random chance and 1 represents maximum separation. IBI, inter-burst interval; rEEG, range-EEG; LM, lower margin; UM, upper margin; SP, spectral power; FD, fractal dimension. Frequency bands: f1, 0.5–3 Hz; f2, 3–8 Hz; f3, 8–15 Hz; and f4, 15–30 Hz.

This first example examines the importance of selecting the right feature set. We compared quantitative features in different EEG vigilance states in preterm infants <32 weeks of gestation. The EEG of preterm infants at this age cycles between two different states of EEG activity. Discontinuous activity consists of short-duration, high-amplitude bursts intermixed with periods of low-amplitude activity, known as inter-bursts; continuous activity consists of mixed frequency activity, without bursts or inter-burst periods. Figure 3 shows an example to illustrate the large differences between these two types of EEG activity.

To be of use, quantitative analysis should be able to capture the complexity of the preterm EEG, and thus should discriminate between segments of discontinuous and continuous activity. Standard deviation of the EEG, the first example feature we presented in the previous section, would not be a useful feature, as there is little difference between the two values (37 and 53 μ*V*) for the two visually different epochs in Figure 3.

We used fully anonymized EEGs from 21 preterm infants recorded within the first few days of birth and selected 2–3 epochs of well-defined continuous activity, and 2–3 epochs of well-defined discontinuous activity, from each EEG record. Each epoch was selected to be artifact free and was approximately 60 s in duration. The median gestational age of the infants was 27.7 weeks, ranging from 25.1 to 31.3 weeks of gestation.

We then estimated qEEG features on each epoch, taking the median across the 2–3 segments for each infant for each activity type. The feature set included spectral power, relative spectral power, interhemispheric coherence, spectral edge frequency, fractal dimension, the median, lower-margin, and upper margin of the rEEG (54). Absolute and relative spectral power, in addition to coherence, were estimated in the four frequency bands: 0.5–3, 3–8, 8–15, and 15–30 Hz (39, 44); rEEG was estimated in 1–20 Hz bandwidth. We also included a burst detection algorithm to estimate the burst ratio and maximum IBI (39). This feature set was introduced in the previous section and in Figure 2. We then calculated the area under the receiver operator characteristic (AUC), with 95% confidence intervals (CIs) using bootstrapping with 1,000 iterations.

The features were ranked according to AUC in Figure 3C. Maximum IBI and burst-ratio were the most discriminating features—not surprising given that discontinuous activity is defined by the presence of bursts and inter-burst. Features of the range EEG (median and lower-margin) also performed well, indicating that the peak-to-peak analysis of the EEG is a good discriminating factor for the two activity types. Lower down the list is spectral power, with relative spectral power even lower. We concluded from this experiment that multiple features, in this case features of the temporal organization of the EEG, are relevant to this specific application. Whereas, the more common features such as spectral power (absolute and relative) and spectral edge frequency are less useful at describing the visually obvious differences between EEG activity types.

## 4. Example 2: Maturation and qEEG

To highlight again the importance of the right feature for the right application, we show how some features are representative of increasing maturation of the preterm brain, whereas other features do not quantify this change. For this we use the same feature set from the preceding example, which includes spectral measures (absolute and relative powers, coherence, fractal dimension, and spectral edge), connectivity (interhemispheric coherence), measures of peak-to-peak amplitude (features of the range EEG), and measures of the burst-inter-burst pattern (features from the burst annotation).

We then selected a 1 h fully anonymized EEG epoch from 23 preterm infants at 24 h postnatal age. Gestational age ranged from 24 to 31.6 weeks, with a median of 28.4 weeks. These EEGs were determined to be appropriate for gestational age, without abnormalities and free from major artifacts (44). The infants had a normal clinical course, without significant adverse events or medication likely to alter the EEG at this time point. An algorithm was used to identify and remove segments of the EEG epoch with artifacts, such as movements or electrode problems (54).

**Figure 4:**
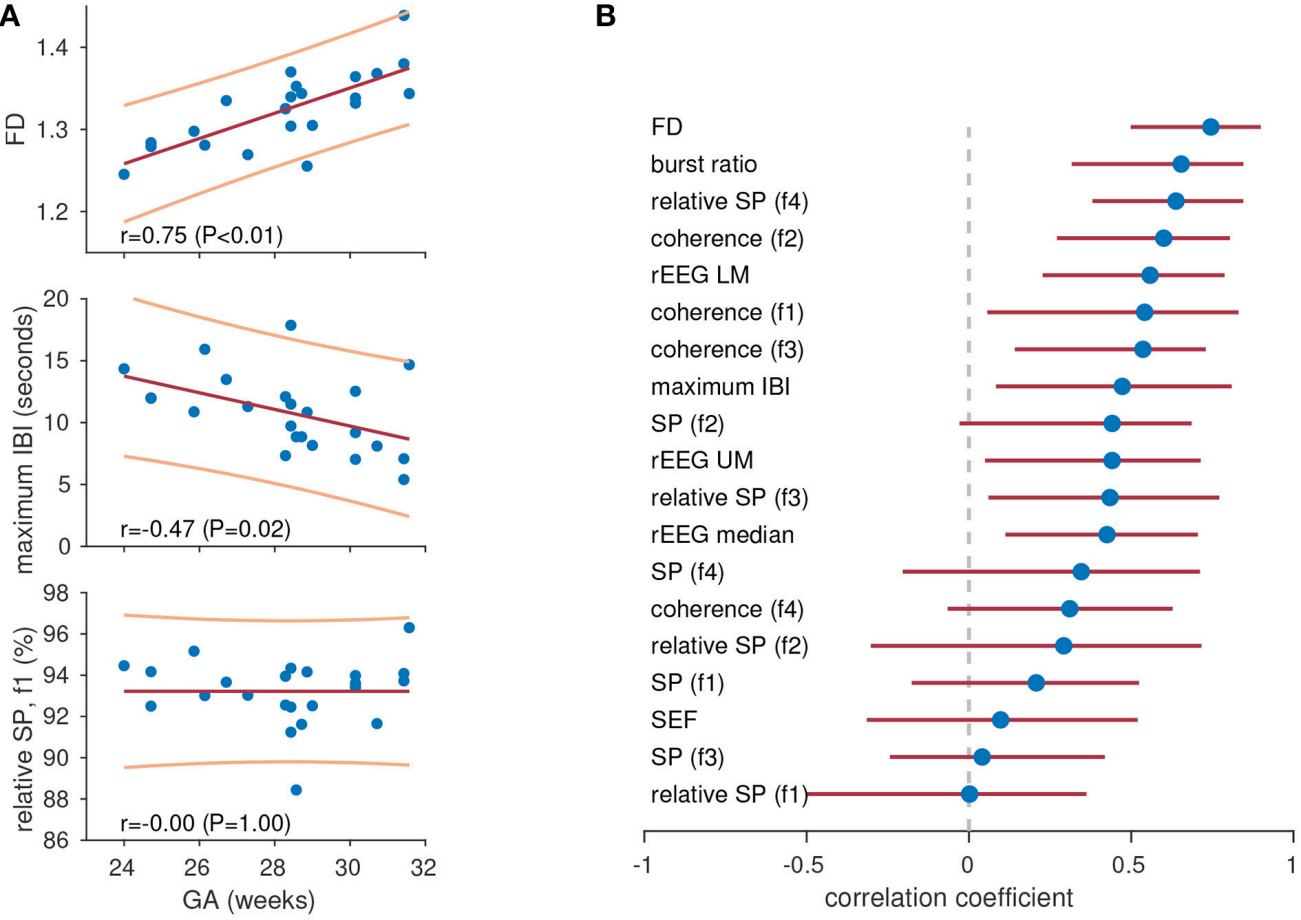
Tracking maturation with quantitative features. Three features in **(A)** showing high (top), medium (centre), and low (bottom) levels of correlation with gestational age (GA). Lines represent least-squares fit (red, centre) with 95% prediction intervals (yellow, top and bottom). Plots show features for 1-h epochs of EEG (dots) in 23 preterm infants. Pearson's correlation coefficient for all features in **(B)**. Dots represents Pearson's correlation coefficient and lines represent 95% confidence intervals. IBI, inter-burst interval; rEEG, range-EEG; LM, lower margin; UM, upper margin; SP, spectral power; FD, fractal dimension. Frequency bands: f1, 0.5–3 Hz; f2, 3–8 Hz; f3, 8–15 Hz; and f4, 15–30 Hz.

The feature set was generated from the 1 h epochs and each feature was correlated with gestational age. We used gestational age as a marker for functional brain maturation, as functional activity is linked to growth (maturation) of the preterm brain (55). We generated Pearson's correlation with 95% CI for each feature, using bootstrapping to generate the CIs. Features are ranked accorded to correlation in Figure 4B, and we observed varying degrees of performance for the different features. Note that the highest ranking features in Figure 3C for the previous application are not always the same features ranked highly in Figure 4B. For example, fractal dimension is the highest (1st) ranking feature in Figure 4B but the lowest (last) in Figure 3C. Again, the common spectral power measures (absolute and relative) are not the highest ranking features. Figure 4A shows an example of significant correlation between the features and gestational age for both the fractal dimension feature and the maximum inter-burst interval features. Figure 4A also shows how the 0.5–3 Hz relative spectral power is, for this example, completely independent (|*r*| < 0.01) of gestational age.

## 5. Example 3: Artifact and EEG

In this last example we examined the effects of artifacts on the qEEG. Artifacts can be biological in origin, such as muscle movement or ECG, and non-biological in origin, such as electrical interference or changing electrode impedance; a more thorough description of common neonatal EEG artifacts can be found elsewhere (56, 57). In a clinical setting it is difficult to record neonatal EEG without encountering some artifacts. Typically, the quantity of artifacts increases with duration of EEG monitoring and the frequency of clinical or routine interventions. The purpose of this example is to show how the qEEG features change when EEG becomes contaminated with artifacts.

**Figure 5:**
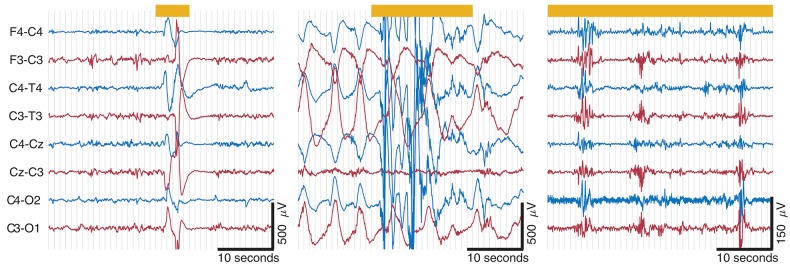
Examples of artifacts taken from EEG records from 3 term infants. Thick yellow line at top represents artifact annotation. Artifact is present across all channels in **(A,B)**; artifact in **(C)** is located in C4-O2, C3-O1, and F4-C4. Note that both **(A,B)** have the same amplitude scaling; high-amplitude slow waves in **(B)** outside of artifact annotation is from a seizure. Movement artifact in **(A,B)** and muscle artifact on C4-O2 in **(C)**. Also ECG artifact present on C3-O1 and F4-C4 in **(C)**.

We examined the fully anonymized EEG from 38 term infants recorded in the NICU within days after birth. Artifacts were annotated on a channel-by-channel basis and included artifacts from cardiac and respiratory sources, artifacts caused by movement and muscle activation, and artifacts caused by poor electrode contact. Examples of different artifacts are presented in Figure 5. This cohort was used in a previous study to develop an automated artifact detection system (58). For each EEG recording from each infant, one epoch of artifact-free EEG was selected and combined with the artifact to build varying percentages of artifact-to-EEG. Specifically, we generated epochs with 15, 30, 60, and 100% of the duration consisting of artifacts.

**Figure 6:**
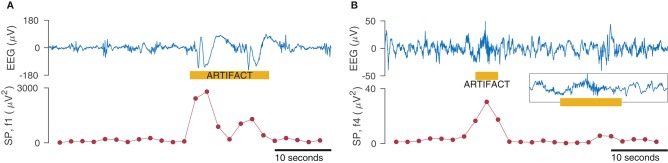
Effect of artifacts on spectral power (SP) measures for the frequency bands 0.5–3 Hz (f1 in **A**) and 15–30 Hz (f4 in **B**). SP is calculated within a 4 s window with 50% overlap for the 50 s EEG (1-channel only). Inset in **(B)** shows zoom of EEG artifact.

A set of qEEG features were computed for each epoch, consisting of spectral power (absolute and relative), spectral edge frequency, fractal dimension, skewness of the EEG, and 3 rEEG features (mean, lower- and upper-margins). The spectral power and EEG skew features were computed for four frequency bands: 0.5–4, 4–7, 7–13, and 13–30 Hz; the rEEG was computed on the 1–20 Hz band. Before generating the feature set, the EEG was low-pass filtered to 30 Hz and then downsampled to 64 Hz. Figure 6 shows how a feature (spectral power at different frequency bands) changes over time in response to an artifact, thus highlighting the deleterious nature of the presence of artifacts.

**Figure 7:**
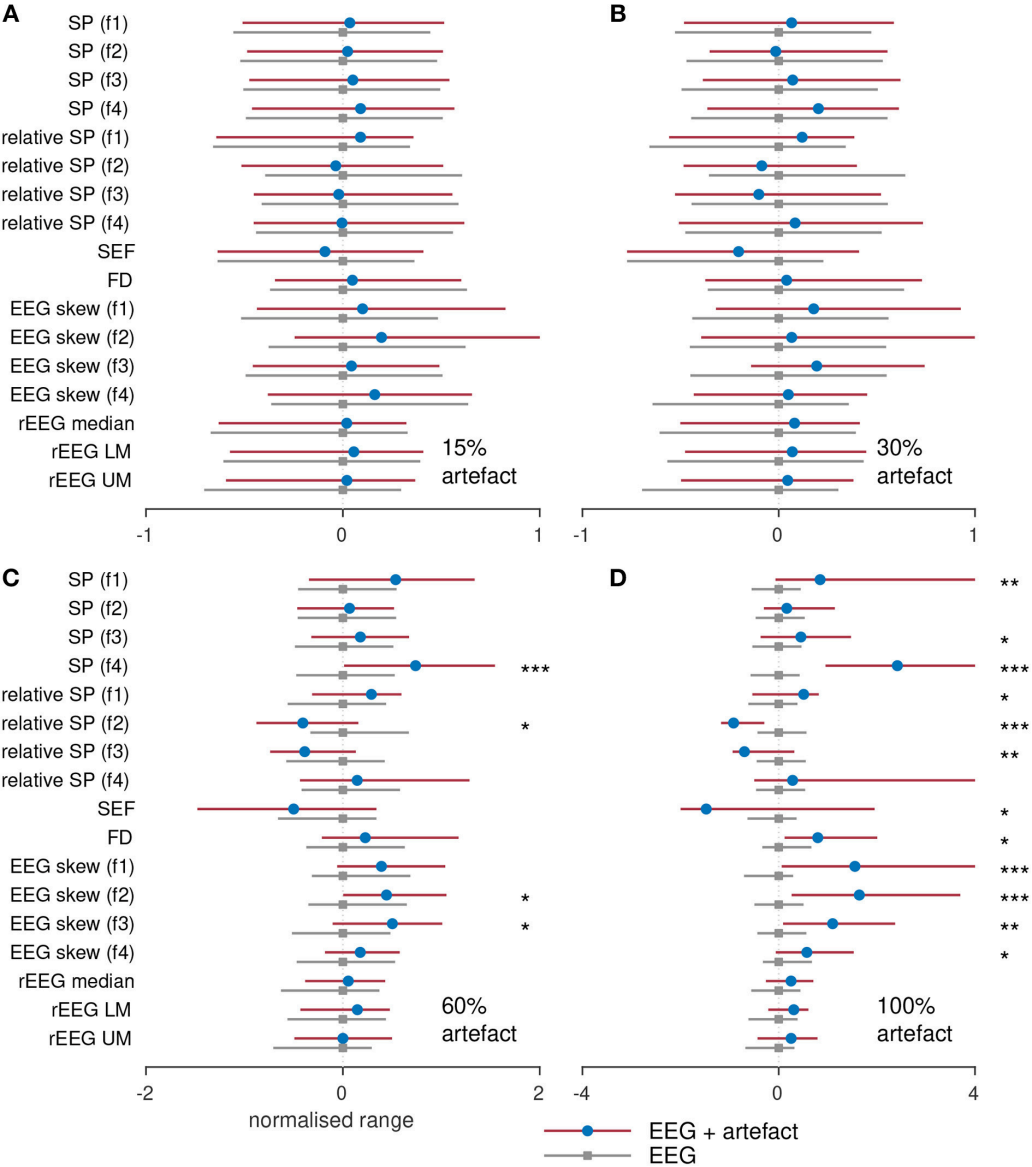
Comparing features with and without varying degrees of artifact. Features generated from 38 term infants EEG with **(A)** 15%, **(B)** 30%, **(C)** 60%, and **(D)** 100% artifacts. SP, spectral power; FD, fractal dimension; SEF, spectral edge frequency; rEEG, range-EEG; LM, lower margin; UM, upper margin; Frequency bands: f1, 0.5–4 Hz; f2, 4–7 Hz; f3, 7–13 Hz; and f4, 13–30 Hz. Stars denote statistical significance from Mann–Whitney tests: ***P < 0.001, **P < 0.01, and *P < 0.05 comparing between EEG epochs and EEG epochs with artifacts. Dots represent median values and lines represents inter-quartile range.

To assess the influence of artifacts on the estimation of the features, we compare the segments of each epoch without artifacts to the epoch containing a percentage of the epoch. For example, we compared the spectral power generated using an artifact-free epoch to the spectral power generated from an epoch containing 10% artifact. These results are plotted in Figure 7 for the four different percentage of artifacts. To plot all features side-by-side, the features are normalized by subtracting the median and dividing by the interquartile range of the feature estimated on the artifact-free epoch. Figures 7A,B show that there is no difference for all features between the epochs without artifacts and the epochs containing 15 and 30% artifacts. In Figure 7C, only 4/17 features significantly (*P* < 0.05) differ for the different epochs. But in Figure 7D, which compares the EEG to the artifact (100% artifact), we see greater differences with 12/17 significant features. This last result is as expected: qEEG features generated from artifacts will differ to the features generated from non-artifact EEG. Although what may be of concern is that the 4–7 Hz spectral power feature, the 13–30 Hz relative-spectral power feature, and none of the rEEG features differed between the EEG and artifacts. This implies that these features are incapable of distinguishing between cortical activity generated EEG and non-cortical activity generated EEG. For the other features, we could say that these features are robust to EEG contaminated with a large percentage of artifacts, in some cases up to 30% or even 60%. Or we could say that because the majority (60%) of the EEG is artifact in Figure 7C, only a few features differ significantly to the artifact-free EEG, thus indicating that these features are incapable of distinguishing between EEG and artifact.

## 6. Procedure for Computing qEEG

**Figure 8:**
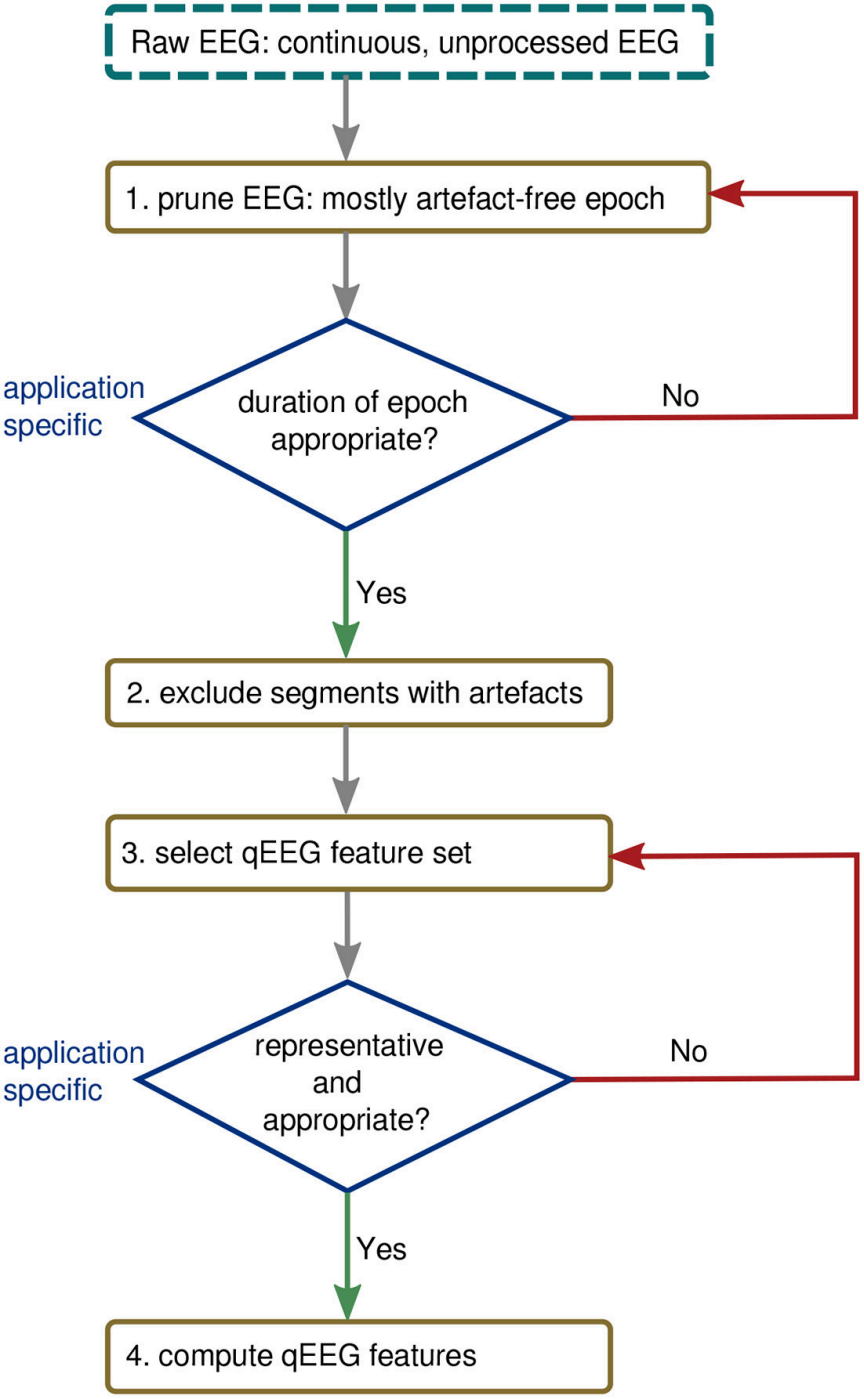
Procedure for generating qEEG from continuous EEG.

Figure 8 presents a process for generating qEEG considering the issues explored in the previous three examples. The first step in Figure 8 requires an experienced clinical neurophysiologist to prune an epoch from the total EEG recording. This epoch should be as free as possible from contaminating artifacts and also be of appropriate duration for the application. The second step acknowledges that there will often be some artifacts still present in the EEG and that these segments of the epoch should be excluded from further analysis. This can be achieved either manually, from visual inspection by the EEG expert, or using an automated procedure, such as existing artifact-detection algorithms (54, 58). The third step should stress the importance of selecting the right feature set for the application: there is no generic feature that will be optimal for all applications in preterm EEG. For example, as the results in Figure 3 show, spectral power does not sufficiently describe the EEG when distinguishing between different activity states nor is it sufficient to estimate functional maturation of the EEG.

## 7. Discussion

Quantitative EEG is a powerful tool for the objective analysis of the EEG. Artifacts have a detrimental effect on the qEEG and should be minimized. Many features are sensitive to maturation and activity states and both should be considered before analysis. Different applications will require different feature sets, and common features such as spectral power or relative spectral power are not sufficient to represent the complexity of the preterm EEG.

As we have shown, it is important that the EEG is free from artifact or contains only minimal artifact. At the very least, it is essential to be aware of the quantity and nature of this artifact before qEEG analysis. This requires human intervention: an EEG expert must review the EEG and select segments that are relatively artifact-free before computing the qEEG features. In the future, automated methods may be able to replace this step (58–60), but to-date none exist for preterm EEG. Even when an expert prunes a segment of EEG for analysis, some artifacts are unavoidable and in this instance it may be helpful to remove periods of artifacts using a simple rule-based procedure (54) or more sophisticated algorithms (60).

Although artifact is a consequence of EEG monitoring in an intensive care environment, there are specific challenges unique to the preterm EEG. First, the epoch used to generate the qEEG must either be long enough, such as 1 h or more, to encompass different states; or, care must be taken to ensure that the EEG activity states are similar between comparison groups. We have demonstrated in section 3 that for many qEEG features, there is a clear separation between activity states. Second, as highlighted in section 4, many features are dependent on gestational age; this extends to maturation in general as there is also a similar progression for postmenstrual age when the EEG monitoring is beyond the first days of life (13–26). This relation is so strong that a combination of qEEG features can be used to predict gestational age with a high level of accuracy (30, 44, 45). Thus, in studies comparing between different preterm groups it may be necessary to control for gestational or postmenstrual age. And third, a recent study has quantified the impact of postnatal adaption to extrauterine life on the qEEG (30). Increased cerebral perfusion during this period may significantly alter cortical activity and thus the qEEG. This study finds an increase in cortical activity, as measured by the qEEG, over the first 3 days of life that is similar to 2–6 weeks of intra-uterine maturation (30). Thus just as gestational age is integral to the assessment of preterm EEG, postnatal age (hours after birth) is also an important factor for preterm qEEG. Postnatal age over the transitional period should also be considered in addition to gestational age.

And lastly, two other important considerations for qEEG are worth mentioning. The first is the problem of how to define the qEEG feature set. There is no one-size-fits-all solution to this problem, as there is an ever-increasing array of statistical and signal processing features that could be considered as qEEG features. The best approach to tackling this problem is to focus on the specific application: what feature set best represents the EEG epochs for this problem? For example, in Figure 4 we see that fractal dimension is the best feature for estimating gestational age whereas it is the worst feature for distinguishing between discontinuous and continuous activity (Figure 3). In addition, it is better to include a large array of qEEG features instead of one or two, such as the commonly used spectral power and relative spectral power measures. A handful of measures may not be sufficient to represent the complex, dynamic preterm EEG. For example, the fractal dimension feature in Figure 4 has the highest ranking correlation with gestational age of *r* = 0.75, however when multiple features are combined using machine learning methods this correlation has been reported to be as high as *r* = 0.89 (44) and *r* = 0.94 (45).

The second problem is that there is no standard for how qEEG features are defined. As most publications do not provide the computer code to generate the features or provide enough details to implement the features, this presents an obstacle to reproducible research. But possibly more troublesome is that the lack of a standard for qEEG makes it difficult to compare results across different studies. We have made an attempt to address this problem by producing open and freely available computer code (53, 61), with full implementation details (54), to define a standard for some of the most common qEEG features. This can only succeed, of course, if the research community engages on this issue.

In conclusion, qEEG in preterms has been successfully applied to many applications of clinical relevance, such as tracking functional maturation, detecting, and even predicting early brain injury, and predicting long-term outcome. We have outlined some key issues that should be considered before implementation, such as artifacts, use of representative epochs, appropriate feature sets for the right application, and the advantages associated with a standard qEEG implementation. Future directions could include the development of an automated scoring system of the preterm EEG using qEEG, a system to quantify the level of abnormalities in the EEG with application to grading EEGs and predicting long-term neurodevelopmental outcome. Clearly, there are many problems to solve before qEEG can be used for routine preterm EEG analysis and more specifically during the vulnerable transition period.

## Author Contributions

GB and JO made substantial contributions to the conception and design of this paper work. GB and JO drafted and revised the paper extensively. Both authors approved this work for publication. Both authors agree to be accountable for all aspects of the work in ensuring that questions related to the accuracy or integrity of any part of the work are appropriately investigated and resolved.

### Conflict of Interest Statement

The authors declare that the research was conducted in the absence of any commercial or financial relationships that could be construed as a potential conflict of interest.
